# Toll-Like Receptors and Dental Mesenchymal Stromal Cells

**DOI:** 10.3389/froh.2021.648901

**Published:** 2021-04-16

**Authors:** Oleh Andrukhov

**Affiliations:** Competence Center for Periodontal Research, University Clinic of Dentistry, Medical University of Vienna, Vienna, Austria

**Keywords:** dental mesenchymal stromal cells, toll-like receptors, differentiation, immunomodulation, lipopolysaccharide

## Abstract

Dental mesenchymal stromal cells (MSCs) are a promising tool for clinical application in and beyond dentistry. These cells possess multilineage differentiation potential and immunomodulatory properties. Due to their localization in the oral cavity, these cells could sometimes be exposed to different bacteria and viruses. Dental MSCs express various Toll-like receptors (TLRs), and therefore, they can recognize different microorganisms. The engagement of TLRs in dental MSCs by various ligands might change their properties and function. The differentiation capacity of dental MSCs might be either inhibited or enhanced by TLRs ligands depending on their nature and concentrations. Activation of TLR signaling in dental MSCs induces the production of proinflammatory mediators. Additionally, TLR ligands alter the immunomodulatory ability of dental MSCs, but this aspect is still poorly explored. Understanding the role of TLR signaling in dental MSCs physiology is essential to assess their role in oral homeostasis, inflammatory diseases, and tissue regeneration.

## Toll-Like Receptors

Toll-like receptors (TLRs) are a family of proteins that play a key role in recognizing pathogens by the innate immune system [1, 2]. TLRs are type I transmembrane proteins consisting of extracellular leucine-rich repeats (LRR) and intracellular toll/interleukin (IL)-1 receptor domains. To date, 10 different TLRs were described in humans. Some of them, particularly TLR-1, TLR-2, TLR-4, TLR-5, TLR-6, and TLR-10, are expressed on the cell surface, whereas TLR-3, TLR-7, TLR-8, and TLR-9 are present only in intracellular compartments such as lysosomes, endosomes, and endoplasmic reticulum [3].

Most TLRs function as homodimers, and only TLR-2 acts as a heterodimer with either TLR-1 or TLR-6 [4]. The crystal structure of the extracellular LRR domain is established for several TLRs [5]. This domain comprises 19–25 tandem LRR copies and contains hydrophobic residues spaced at specific intervals [5, 6]. Various human TLRs differ in the number of LRR and domain structures, leading to recognizing different ligands [5]. Each TLR recognizes specific, highly conserved bacterial or viral structures. These structures are common for various pathogens and are crucial for their function. The most known TLR ligands are bacterial lipopeptides (TLR-2/TLR-1 and TLR-2/TLR-6), viral double-stranded RNA (TLR-3), lipopolysaccharide (TLR-4), bacterial flagellin (TLR-5), bacterial or viral single-stranded RNA (TLR-7 and TLR-8), and CpG-rich unmethylated DNA (TLR-9) [7]. The ligand and function of TLR-10 are poorly known [8]. It was shown that, in contrast to other TLRs, TLR-10 has an anti-inflammatory action and dampen TLR-2 response [9]. A recent study indicated that TLR-10 might sense HIV-1 envelope protein [10]. Besides exogenous ligands, TLRs might also be activated by endogenous ligands released from damaged tissue or dead cells [11]. Activation of TLRs by endogenous ligands has a crucial role in the regulation of local tissue homeostasis.

After ligand binding, the intracellular TIR domain recruits adaptor molecules, which initiate the response [2]. The following adaptors are described: myeloid differentiation primary response gene 88 (MyD88), TIR-domain-containing adaptor protein (TIRAP), TIR domain-containing adaptor inducing interferon-β (TRIF), and TRIF-related adaptor molecule (TRAM) [12]. Activation of almost all TLRs excepting TLR-3 results in triggering of MyD88- or MyD88/TIRAP-dependent response, leading to the activation of nuclear factor kappa B (NF-κB) and proinflammatory cytokine production. Ligand binding to TLR-3 activates the TRIF-dependent pathway and induces type I interferon (IFN) signaling. Finally, TLR-4 activates both NF-κB mediated by MyD88 and type I IFN pathway through TRAM/TRIF [3].

## Dental Mesenchymal Stromal Cells

The International Society for Cell and Gene Therapy (ISCT) defines mesenchymal stromal cells (MSCs) as plastic-adherent fibroblast-like cells; expressing mesenchymal surface markers CD73, CD90, and CD105; lacking hematopoietic surface markers CD11b, CD14, CD34, CD45, and HLA-DR; and possessing the ability to differentiate into osteoblasts, adipocytes, and chondrocytes *in vitro* [13, 14]. For the first time, MSCs were isolated from the bone marrow, but later, MSCs were found in almost all postnatal tissues [15], including dental pulp [16], human exfoliated deciduous teeth [17], periodontal ligament [18], apical papilla [19], dental follicle [20], gingival tissue [21], and periapical cyst [22]. Most dental-tissue-derived MSCs also express several neural lineage markers, presumably due to their neural crest origin [23–25]. Sometimes, the abbreviation “MSCs” is used as an acronym for “mesenchymal stem cells.” There is an ongoing discussion if these cells should be classified as “stromal” or “stem” cells mainly because of their limited differentiation ability *in vivo* and lacking asymmetric division [26, 27]. In the present review, we will adhere to the recent recommendation of ISCT to use the term “mesenchymal stromal cells” in combination with tissue origin [14].

Despite the high plasticity of MSCs *in vitro*, the differentiation ability of transplanted MSCs *in vivo* is very limited [28]. Nowadays, there is a large consensus that the therapeutic effect of MSCs is achieved through either secretion of specific trophic factors or immunomodulatory function [29]. As reviewed by several papers, dental MSCs possess a strong immunomodulatory ability and can regulate the function of different immune cells [30–35]. The effects of dental MSCs are most often immunosuppressive and are mediated by the production of soluble factors and direct cell-to-cell contact [30]. The immunomodulatory capacity of dental MSCs is usually low and is boosted by different inflammatory cytokines like IFN-γ, tumor necrosis factor (TNF)-α, and interleukin (IL)-1β. These cytokines are produced by the activated immune cells and upregulate the expression of different immunomodulatory factors in dental MSCs, e.g., indolamine-2,3-dioxygenase 1 (IDO-1), prostaglandin E2 (PGE2), TNF-α-stimulated gene 6 (TSG-6), programmed cell death-ligand 1 (PD-L1), and PD-L2 [36–40]. Thus, it seems that dental MSCs and immune cells regulate each other's activity in a reciprocal manner, which can be implicated in various processes such as tissue regeneration and inflammatory disease progression [30].

According to ISCT criteria, the cell population can be defined as MSCs if more than 95% of the population expresses mesenchymal markers and <2% of the population expresses hematopoietic markers. However, despite these strict criteria, MSCs usually represent rather heterogeneous than homogeneous cell population [41]. Considerable heterogeneity is observed even within single-cell-derived MSCs clones [42]. Besides MSCs themselves, cell populations might comprise osteoblasts, fibroblasts, and other cells of mesenchymal origin, which surface markers are indistinguishable from those of MSCs [43, 44]. It should be noted that fibroblasts-like cells were isolated from various human dental tissues, e.g., dental pulp (human dental pulp cells, hDPCs), gingiva (human gingival fibroblasts, hGFs), and periodontal ligament (human periodontal ligament cells, hPDLCs). These cells share many properties of the corresponding “stem cell” populations isolated from these tissues and express similar surface markers [45–47]. The present review will comprise the studies with both MSC-like cells and fibroblast-like cells from various dental tissues, and the cell names will be indicated as they are mentioned in the corresponding paper.

Toll-like receptors impact MSCs biology and affect their functions such as proliferation, migration, differentiation potential, immunomodulatory ability, and survival [48–50]. The oral cavity is a habitat for different microorganisms [51, 52]. Host–microbial homeostasis is a crucial determinant of oral health, and its disruption is associated with oral diseases, like caries and periodontitis [53]. Interaction of bacteria- and viral-derived TLR ligands might affect the functional properties of dental MSCs and needs to be understood. This narrative review aims to summarize state-of-the-art on the role of TLRs and their ligands in dental MSCs.

## TLRs Expression in Dental MSCs

The expression of TLRs in different dental MSCs was investigated specifically rather rarely. However, the presence of some TLRs, like TLR-2, TLR-3, and TLR-4 in these cells is indisputable because of their responsiveness to the corresponding ligands [54–56]. Some studies investigated specifically the expression and regulation of TLRs in various dental MSCs. Li et al. analyzed the expression of different TLRs in human periodontal ligament stem cells (hPDLSCs) and compared it with that in bone marrow MSCs [57]. They found that in comparison to BM-MSCs, hPDLSCs express significantly higher levels of TLR-1, TLR-2, and TLR-5, as well as significantly lower levels of TLR-3, TLR-4, TLR-6, TLR-8, TLR-9, and TLR-10 [57]. Zhu et al. showed that hPDLSCs express TLR-1, TLR-2, TLR-3, TLR-4, and TLR-6 on both gene and protein levels as shown by quantitative PCR (qPCR) and flow cytometry analysis, respectively [58]. El-Sayed et al. investigated the expression of TLRs in different dental MSCs population and its regulation by the inflammatory cytokine cocktails consisting of IL-1β, IFN-α, IFN-γ, and TNF-α [59, 60]. Human gingival MSCs (hGMSCs) were shown to express TLR-1, 2, 3, 4, 5, 6, 7, 10 on the gene and protein levels [59]. Treatment of these cells with the inflammatory cocktail resulted in the down-regulation of TLR-6 and upregulation of all other TLRs [59]. Resting human dental pulp stem cells (hDPSCs) were found to express all TLRs in different quantities [61]. Treatment of DPSCs with inflammatory cytokines induced upregulation of TLR-2, 3, 4, 5; downregulation of TLR-1, 7, 9, 10; and abolishment of TLR-6 [61]. MSCs derived from alveolar bone were found to express all TLRs except TLR-9 [60]. The highest expression levels were found for TLR-2 and the lowest one for TLR-6 [60]. Thus, there are some differences in the TLRs expression and regulation between dental MSCs of different origins, but their physiological importance for a particular specific tissue should still be established.

## Effect of TLRs Ligands on Differentiation Potential of Dental MSCs

Numerous studies investigated the effect of TLR ligands on the differentiation potential of dental MSCs *in vitro*. The majority of them focused on the impact of lipopolysaccharide (LPS) on osteogenic differentiation, presumably because of the putative role of dental MSCs in alveolar bone regeneration. In these studies, osteogenic differentiation was assessed by the expression of specific markers like alkaline phosphatase (ALP), osteocalcin (OCN), collagen 1 (Coll-1), osteopontin (OPN), osterix (OSX), and runt-related transcription factor 2 (RUNX-2) as well as by mineralization assay (alizarin red staining). LPS was used as an essential virulence factor of Gram-negative bacteria, which is involved in the etiology of periodontitis and pulpitis [62, 63].

In contrast to LPS, the effect of other bacterial components on the osteogenic differentiation of dental MSCs is investigated rarely. However, such studies would be especially important because both Gram-negative and Gram-positive bacteria continuously secrete numerous proteins, which might activate various TLRs and exert multiple cellular effects in MSCs. Particularly, lipoteichoic acid, peptidoglycan, and fimbriae activate TLR-2 [4, 64–66]; bacterial flagellin activates TLR-5 [67]. A microarray study showed that *Porphyromonas gingivalis* secreted products activated several signaling pathways involved in bone metabolism and inflammatory and immune response [68], and therefore, identifying the potential contribution of all TLRs in dental MSCs physiology would be very important [67].

### Periodontal-Ligament-Derived MSCs

The effect of LPS on the osteogenic differentiation of periodontal-ligament-derived MSCs is investigated most extensively. Li et al. showed that *Escherichia coli* LPS (10 μg/ml) decreases osteogenic differentiation and RUNX-2 expression in human periodontal ligament stem cells (hPDLSCs) but not that of BM-MSCs [57]. This effect was mediated through TLR-4 induced NF-κB activation [57]. Kato et al. reported that *P. gingivalis* LPS (1–10 μg/ml) inhibits mineralization and expression of ALP, OCN, and Coll-1 by human hPDLSCs [69]. Wei et al. found that *E. coli* LPS (10 μg/ml) inhibits osteogenic differentiation, alkaline phosphatase expression and activity, and gene expression of OCN, Coll-1, and RUNX-2 in hPDLCs [70]. Kim et al. demonstrated that *E. coli* LPS (2 μg/ml) inhibits osteogenic differentiation of hPDLCs and the expression of BMP-2, OSX, and RUNX-2 [71]. Zhu et al. showed that *E. coli* LPS (1–10 μg/ml) inhibits ALP activity and mineralization of hPDLSCs, and this effect was partially reversed by MyD88 and TRIF silencing [58]. Wang et al. reported that *E. coli* LPS (0.1–10 μg/ml) inhibits osteogenic differentiation, ALP activity, and RUNX-2 expression of hPDLSCs through TLR-4 dependent mechanism [72]. Yu et al. showed that *P. gingivalis* LPS (10 μg/ml) inhibits the osteogenic differentiation of hPDLSCs and decreases the expression of OCN, RUNX-2, and Coll-1 [73]. Blufstein et al. found that *P. gingivalis* LPS (1 μg/ml) in combination with soluble CD14 inhibits the basal and vitamin-D_3_-induced expression of OCN and OPN in hPDLCs [74].

Some studies did not confirm the inhibitory effect of LPS on the osteogenic differentiation of periodontal-ligament-derived MSCs. Jönsson et al. showed that *E. coli* LPS (0.5–10 μg/ml) does not affect Coll-1 production by hPDLCs [75]. Li et al. did not find any significant effect of *E. coli* LPS (1 μg/ml) on the osteogenic differentiation, ALP activity, gene and protein expression of ALP, RUNX-2, and Coll-1 by hPDLCs [76]. Albiero et al. did not observe any influence of *P. gingivalis* LPS (1 μg/ml) on the osteogenic potential of hPDLSCs [77]. Jia et al. reported that *P. gingivalis* LPS (10 μg/ml) does not affect the expression of ALP, Coll-1, RUNX-2, OCN, OPN, and OSX in hPDLCs but inhibits it when the cyclic stress was applied to the cells [78].

Some studies reported the stimulatory effect of LPS on osteogenic differentiation. Albeiro et al. showed that *E. coli* LPS (1 μg/ml) stimulates osteogenic differentiation as well as the expression of ALP, OCN, and RUNX-2 [79]. Xing et al. observed that *E. coli* LPS (0.5 μg/ml) stimulates osteogenic differentiation, ALP activity, and the expression of ALP, RUNX-2, OCN, and Coll-1 presumably through Wnt/β-catenin-dependent mechanism [80]. Thus, it seems that the effect of LPS on the osteogenic differentiation of periodontal-ligament-derived MSCs depends on the concentration and, to a lesser extent, on LPS source. High LPS concentrations inhibit osteogenic differentiation, whereas low LPS concentrations have no effect or even stimulate it.

Only two studies addressed the effect of other TLRs ligands on the osteogenic potential of periodontal-ligament-derived MSCs. Zhu et al. found that TLR-2/1 ligand Pam3CSK4 and TLR-2/6 ligand FSL-1 inhibit mineralization and ALP activity of hPDLSCs in a concentration-dependent manner, and this effect was partially reversed after MyD-88 knockdown [58]. TLR-3 ligand Poly I:C enhanced osteogenic differentiation and ALP activity at low concentration (0.1 μg/ml) and inhibited these parameters at higher concentration (10 μg/ml) [58]. Blufstein et al. showed that both basal and vitamin-D_3_-induced expression of OCN and OPN are inhibited by TLR-2/1 ligand Pam3CSK4 [74].

Besides participating in alveolar bone metabolism, periodontal-ligament-derived MSCs participate in the cementogenesis [81]. Kim et al. showed that *E. coli* LPS (2 μg/ml) inhibited the expression of CEMP-1, which is involved in cementogenesis [71].

### Dental-Pulp-Derived MSCs

Some contradictory data are reported regarding the effect of LPS on osteogenic differentiation of dental-pulp-derived MSCs. Yamagishi et al. found that *P. gingivalis* LPS (5–20 μg/ml) induces a dose-dependent inhibition of OCN expression in hDPSCs [82]. Yuan et al. showed that *E. coli* LPS (10 μg/ml) inhibits the mineralization and expression of ALP, OCN, OPN, OSX, and RUNX-2 in rat dental pulp stem cells [83]. In contrast, Huang et al. reported a dose-dependent increase in ALP activity and mineralization of human dental pulp cells (hDPCs) by *E. coli* LPS (0.1–10) [84]. He et al. found that the stimulatory effect of *E. coli* LPS (1 μg/ml) on mineralization of hDPSCs and expression of ALP and OCN is mediated by TLR-4 activation [85]. Chung et al. demonstrated that *P. gingivalis* LPS (1 μg/ml) in combination with soluble CD14 stimulates the expression of OCN and mineralization of hDPSCs [86]. Wildbiller et al. did not observe any significant effect of *E. coli* LPS (0.01–1 μg/ml) on the expression of OCN and Coll-1 in hDPSCs [87].

Besides common trilineage differentiation potential, dental-pulp-derived MSCs can differentiate into odontoblast and assumed to participate in dentin regeneration [88]. The effect of TLR ligands on the odontogenic differentiation of dental-pulp-derived MSCs is differently discussed in the literature. Yamagishi et al. reported that the expression of dentin sialophosphoprotein (DSPP) in hDPSCs is inhibited by *P. gingivalis* LPS (5–20 μg/ml) in a concentration-dependent manner [82]. Wildbiller et al. showed that *E. coli* LPS (0.01–1 μg/ml) suppress the expression of DSPP and dentin matrix protein 1 (DMP-1) in hDPSCs induced by extracted dentine matrix proteins but does not affect their basal expression [87]. Huang et al. found that *E. coli* LPS (0.1–10 μg/ml) enhances the expression of DSPP and DMP-1 in hDPCs in a dose-dependent manner [84]. He et al. showed that *E. coli* LPS induces DSPP and DMP-1 in hDPSCs through the mitogen-activated protein kinase signaling pathway [85]. Finally, the exosomes from LPS preconditioned DPSCs promoted proliferation, migration, and odontogenic differentiation of Schwann cells [89].

### Apical Papilla and Dental-Follicle-Derived MSCs

Apical-papilla-derived MSCs (stem cells from apical papilla, SCAP) reside in the apical papilla of permanent teeth. They possess osteogenic, adipogenic, chondrogenic, neurogenic, and odontogenic differentiation potential [90]. Lei et al. found that *P. gingivalis* LPS (5 μg/ml) inhibited mineralization and expression of ALP, RUNX-2, and DMP-1 in SCAP by inducing autophagy [91]. Kukreti et al. showed that the culture of SCAP on *Pseudomonas aeruginosa*-coated dentin strongly inhibits mineralization and expression of DSPP and DMP-1 [92].

Human dental follicle stem cells (hDFSCs) are isolated from follicle tissue surrounding the tooth germ [20, 93]. Morsczeck et al. found that *E. coli* LPS (1 μg/ml) and *P. gingivalis* LPS (1 μg/ml) stimulates ALP activity but inhibits the mineralization of human hDFSCs [94]. The effect of *E. coli* LPS was more pronounced than that of *P. gingivalis* LPS [94].

### Gingiva-Derived MSCs

Gingiva-derived MSCs are unique MSCs that possess multilineage differentiation potential and are considered to be promising cells for oral tissue regeneration [95]. Karlis et al. showed that TLR-2/1 ligand Pam2CSK4, ultrapure *P. gingivalis* LPS, and standard *P. gingivalis* LPS (all 0.01 μg/ml) do not affect the mineralization (calcium deposition) and the expression of ALP and osteonectin in chronically stimulated human GFs [96]. The same study found that GFs chronically stimulated with TLR-2 and TLR-4 ligands slightly attenuate osteoclastogenesis activity in coculture experiments [96].

## TLR Ligands and the Immunoregulatory Role of Dental MSCs

MSCs produce a plethora of various factors involved in regulating the inflammatory response [97]. The production of these factors is usually upregulated by inflammatory cytokines and TLRs ligands [48, 97]. One group of these factors includes proinflammatory cytokine and chemokines like IL-1β, TNF-α, IL-6, IL-8, MCP-1, etc. These proteins usually have a proinflammatory action, promote immune cell migration, and induced tissue destruction. The second group of factors comprises different immunosuppressive proteins like IDO-1, PGE2, TSG-6, PD-L1, PD-L2, and TGF-β. These proteins have immunosuppressive anti-inflammatory effects related to “immunomodulatory properties of MSCs” [30, 98]. Activation of MSCs with TLR ligands usually activates both proinflammatory and anti-inflammatory responses, and the balance between them depends on the type and concentration of TLR ligand [49].

### The Proinflammatory Response of Dental MSCs to TLR-4 Ligand Lipopolysaccharide

LPS is a cell wall component of Gram-negative bacteria and is a well-known TRL-4 ligand [99]. Numerous studies dealt with the effect of different LPS preparations on the production of various proinflammatory factors by dental MSCs. In these studies, MSC-like cells from various dental tissues were with LPS at concentrations ranging from 0.01 to 50 μg/ml. The resulting production of various proinflammatory factors was detected [e.g., [100, 101]]. For the sake of clearness and due to space limitation reasons, only some critical aspects of LPS-induced response in dental MSCs will be mentioned without the detailed overview of all existing data.

LPS is recognized by TLR-4 in complex with MD-2. The binding of LPS to the TLR-4/MD-2 complex is facilitated by lipopolysaccharide-binding protein and CD14 [102, 103]. The binding of LPS to CD14 enhances the sensitivity of host cells to endotoxin and enables sensing it even at picomolar concentrations [104]. Besides, CD14 is required for the internalization of TLR-4 and activation of TRIF-dependent signaling [105]. Membrane-bound CD14 (mCD14) is a GPI-anchored protein, which is expressed by various immune cells [106]. However, MSCs, by definition, do not express mCD14 on their surface [13]. Besides the membrane-bound form, there is also a soluble form of CD14 (sCD14) [107]. Our group showed that sCD14 increases the sensitivity and amplitude of hPDLSCs to *P. gingivalis* LPS and *E. coli* LPS [100]. sCD14 is present in serum, saliva, and gingival crevicular fluid, and therefore, it might be implicated in dental MSCs response to LPS in *in vivo* situations [108–110]. However, the majority of studies on the effect of LPS on dental MSCs does not use sCD14.

An increase in the production of IL-6, IL-8, and MCP-1 by periodontal-ligament-derived MSCs is most often reported [69, 75, 100, 111–113]. Some studies also reported that LPS increases the production of IL-1β and TNF-α by periodontal-ligament-derived MSCs [70, 71, 79], whereas one study did not confirm this finding [113]. Additionally, a stimulatory effect of LPS on the expression of IL-12, intercellular adhesion molecule 1, vascular adhesion molecule 1, and macrophage colony-stimulating factor is described [71, 113, 114]. The responsiveness of periodontal-ligament-derived MSCs to LPS might be modified by the inflammatory environment. Early studies suggest that hPDLCs are unresponsive to LPS from *E. coli* and *Aggregatibacter actinomycetemcomitans* (0.1 μg/ml) [115]. Pretreatment with a low concentration of IL-1β induced the responsibility of these cells to both LPS [115]. LPS-primed hPDLSCs were shown to promote macrophage polarization toward a proinflammatory M1 phenotype [116].

In gingiva-derived MSCs, bacterial LPS induced the production of IL-6, IL-8, and MCP-1 [117–120]. The data on the production of IL-1β and TNF-α by hGFs upon LPS stimulation are contradictory: it is supported by some studies [121, 122] and denied by other studies [123, 124]. Additionally, GFs produced CCL5 [125], macrophage inflammatory protein-3 upon stimulation with LPS [126]. Pretreatment of hGFs with IFN-γ enhanced the expression of CD14, TLR-2, and TLR-4; induced surface expression of CD14; and increased responsiveness to LPS stimulation [127].

Several studies investigated the proinflammatory response of other dental-derived MSCs to bacterial LPS. *Porphyromonas gingivalis* LPS induced IL-6 and IL-8 and inhibited TGF-β production by hDPSCs [86]. In hDPCs, *E. coli* LPS enhanced the gene expression of IL-6, IL-1β, and TNF-α [128]. Stimulation of SCAP with LPS resulted in the upregulation of IL-6, IL-8, IL-1β, and TNF-α [129, 130]. In DFSCs, both *E. coli* LPS and *P. gingivalis* LPS induced the production of IL-6, IL-8, and MCP1, and the effect of *E. coli* LPS was markedly higher than that of *P. gingivalis* LPS [94]. In another study on dental follicle progenitor cells, *P. gingivalis* LPS could not induce IL-6 production but changed the expression of TLR-2 and TLR-4 and stimulated cell migration [131].

It should be noted that several factors could influence the response of dental MSCs to LPS. Our recent study showed that LPS purity is an essential factor influencing the response of hPDLSCs and hGMSCs to bacterial LPS [119]. Commercially available LPS preparations are usually contaminated by lipoproteins (about 2%). When LPS is applied at a concentration of 50 μg/ml, the concentrations of contaminating lipoproteins might reach 1 μg/ml. At this concentration, lipoproteins can induce a robust inflammatory response [66, 132], and therefore, it is difficult to discriminate if the response originates from LPS or lipoproteins. Contaminating lipoproteins also account for the ability of some *P. gingivalis* LPS preparation to activate TLR-2 response [119, 133]. Stimulation time is another factor influencing the response to LPS. For example, Widbiller et al. showed that *E. coli* LPS does not affect IL-6 production by hDPSCs after a short time (1 day) but increases it after 4–7 days [87].

### The Proinflammatory Response of Dental MSCs to Other TLR Ligands

The effect of TLR-2 and TLR-3 ligands on the production of proinflammatory cytokines by dental MSCs is investigated relatively rarely. Some studies of our group showed that TLR-2/1 ligand Pam3CSK4, TLR-2 ligand lipoteichonic acid (LTA), and TLR-3 ligand Poly I:C induce the production of IL-6, IL-8, and MCP-1 by hPDLSCs by a much greater extent than LPS [56, 66, 100, 134]. The response of hPDLSCs to TLR-2 ligands is enhanced by sCD14 [66], which is not surprising because CD14 serves as an assessor molecule for TLR-2 [103]. Pam3CSK4, Poly I:C, and TLR-2/6 ligand FSL-1 activated NF-kB and increased the gene expression of IL-6, IL-8, TNF-α, and IL-1β in hPDLSCs [58]. In GFs, Poly I:C, FSL-1, TLR-7/8 ligand ssPolyU, and TLR-9 ligand CpG DNA significantly induced the production of IL-6, IL-8, and MCP-1 [135]. In contrast, another study on hGFs showed that IL-8 production was enhanced by LPS, Poly I:C, and TLR-5 ligand flagellin but not by TLR-7, 8, and 9 ligands [136]. Different bacterial LTA and Pam3CSK4 induced IL-6 and IL-8 production in hGFs [137, 138]. TLR-2-primed hGFs stimulated the proliferation of CD3^+^-positive T cells [139].

### Immunomodulatory Activity of TLR-Primed Dental MSCs

The role of different TLRs in the immunomodulatory ability of MSCs in general is still not entirely understood. Earlier studies suggested that the priming of MSCs with TLR-2- or TLR-4-primed MSCs stimulate the immune response, whereas TLR-3-primed MSCs exhibit immunosuppressive properties [49, 140]. However, some studies challenged this conception [141, 142]. Thus, the immunomodulatory role of different TLRs in MSC-mediated immunomodulation still needs to be investigated. Unfortunately, there are only a limited number of studies in which the effects of different TLR ligands on the immunomodulatory activity of dental MSCs are investigated.

Tomic et al. found that TLR-3 ligand poly I:C enhanced the inhibitory effect of MSCs derived from dental pulp and dental follicle on peripheral blood mononuclear cells (PBMCs) proliferation [143]. However, TLR-4 ligand LPS augmented immunosuppression only in dental follicle MSCs and abrogated it in dental pulp MSCs [143]. The anti-TGF-β antibody strongly abrogated the immunosuppressive effect of both cell types. Moreover, the effect of TLR ligands on TGF-β expression showed a similar pattern as for immunosuppressive properties. Similarly, TLR-3 ligand enhanced TGF-β production in both MSCs types, whereas TLR-4 ligand LPS enhanced TGF-β production in dental follicle MSCs and inhibited it in dental pulp MSCs. Priming hPDLSCs with LPS decreased the frequency of CD33^+^ and CD14^+^ myeloid cells within the PBMCs population but did not affect their immunosuppressive activity on T cell proliferation and differentiation [112]. GFs pretreated with *P. gingivalis*-derived LPS stimulated the ability of the GFs to suppress PBMCs proliferation and enhanced the IFN-γ-induced immunosuppressive ability [136]. However, the immunomodulatory effect of *P. gingivalis* LPS was relatively small compared with that of IFN-γ [136].

TLR ligands were shown to affect the expression of some immunomodulatory proteins in dental MSCs. MSC-mediated immunosuppression in humans is largely mediated by indoleamine-2,3-dioxygenase-1 (IDO-1), which catalyzes the catabolism of L-tryptophan into L-kynurenine [144], and the resulting depletion of tryptophan leads to the immunosuppression [145]. The majority of existing reports suggest that TLR ligands enhance IDO-1 gene expression in dental MSCs. The enhanced IDO-1 gene expression was induced in hPDLs by *E. coli* LPS [146]; in hPDLSCs by Pam3CSK4 and Poly I:C [147, 148]; in hDPSCs by *E. coli* LPS [149]; in hGFs by *P. gingivalis* LPS, *E. coli* LPS, and flagellin [40, 136]; and in hGMSCs by Poly I:C [150]. No stimulatory effect on IDO-1 gene expression was observed in hPDLSCs upon *E. coli* LPS stimulation [147] and in hGMSCs upon stimulation with TLR-1, 2, 4, 6, and 7 ligands [150]. In contrast to the gene expression data, the effect of various TLRs ligands on IDO-1 protein expression and enzymatic activity in dental MSCs is somewhat controversial. Intracellular IDO-1 expression was not affected by Pam3CSK4 and *E. coli* LPS in hPDLSCs [147], by *P. gingivalis* and *E. coli* LPS in hDPSCs and hDFSCs [143], and PamsCSK4 and *E. coli* LPS in hDPSCs [39]. In contrast, IDO-1 protein expression was enhanced in hPDLSCs by Poly I:C and in hDPSCs by *E. coli* LPS [149]. The enzymatic activity of IDO-1 in conditioned media was reported to be increased by Pam3CSK4 and Poly I:C in hPDLSCs [148] and by *E. coli* LPS in hPDLs [146]. In contrast, no effect of *P. gingivalis* LPS on IDO-1 activity was observed in GFs [40]. Several studies reported that IDO-1 expression and activity induced by TLR-3 ligand Poly I:C is substantially higher than those induced by other TLR ligands [147, 149, 150]. This observation suggests a superior role of TLR-3 signaling in the immunomodulatory properties of dental MSCs, which should be confirmed by future functional studies.

## Concluding Remarks and Future Perspective

Resident dental MSCs are involved in both inflammatory response and dental tissue repair after trauma. The clinical protocols for applying dental MSCs for the treatment of periodontal and endodontic defects are currently developing [151–153]. Besides, due to their accessibility and functional properties, dental MSCs have an enormous potential for application beyond the dental field [23]. Numerous preclinical researches imply an enormous perspective of dental MSCs for the application in bone and cartilage repair and the treatment of immunological disorders [30, 154, 155]. The mechanisms underlying *in vivo* regenerative potential are based mainly on modifying the environment [30, 156]. Furthermore, the exosomes of dental MSCs are considered to be a promising tool for the regeneration of oral and extraoral tissues [157, 158].

**Figure 1:**
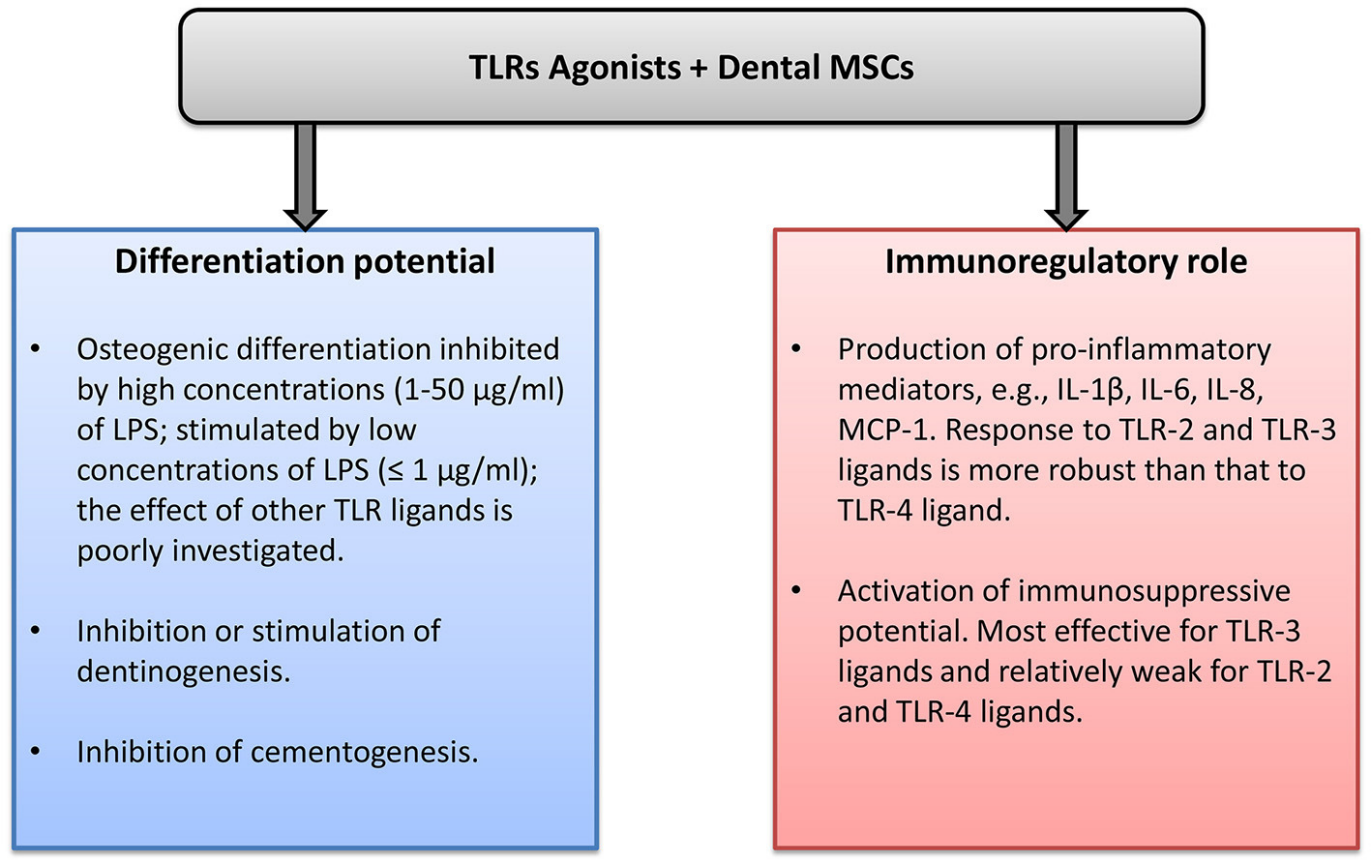
Effects of toll-like receptor (TLR) agonists in dental mesenchymal stromal cells (MSCs).

Dental MSCs express all human TLRs, and the effects of TLRs ligand in dental MSCs are summarized in Figure 1. The differentiation capacity of dental MSCs can be either diminished or enhanced by various TLR ligands. This effect might depend on the concentration and type of TLR ligand. Stimulation of dental MSCs with different TLR ligands induces the production of various proinflammatory mediators, mainly IL-6, IL-8, and MCP-1. This fact suggests that dental MSCs might play an important role in the progression of different inflammatory diseases. However, the exact role of dental MSCs in oral diseases such as pulpitis and periodontitis is still to be clarified. The role of TLRs in the immunomodulation by dental MSCs is investigated rather poorly to date. Some reports suggest that TLR-primed MSCs promote immune response, whereas other reports indicate an immunosuppressive effect of TLR-treated dental MSCs. Future well-designed studies are necessary to clarify the role of TLRs in the immunomodulatory ability of dental MSCs. Understanding the role of MSCs in the inflammatory processes could open new perspectives for dental tissue regeneration and treatment of the inflammatory diseases.

## Author Contributions

OA created the concept and wrote the manuscript.

## Conflict of Interest

The author declares that the research was conducted in the absence of any commercial or financial relationships that could be construed as a potential conflict of interest.
